# Dynamic Change of Lymphocyte-to-Monocyte Is Associated With the Occurrence of POCD After Cardiovascular Surgery: A Prospective Observational Study

**DOI:** 10.3389/fnbeh.2021.646528

**Published:** 2021-04-13

**Authors:** Qi Zhao, Rui Gao, Changliang Liu, Hai Chen, Xueying Zhang, Jing Guan, Xiaoyu Xie, Yanhua Qiu, Xu Cheng, Peilin Lv, Tao Zhu, Chan Chen

**Affiliations:** ^1^Department of Anesthesiology and Translational Neuroscience Center, West China Hospital, Sichuan University, Chengdu, China; ^2^Targeted Tracer Research and Development Laboratory, Department of Respiratory and Critical Care Medicine, West China Hospital, Sichuan University, Chengdu, China

**Keywords:** postoperative cognitive dysfunction, Lymphocyte-to-monocyte ratio, cardiovascular surgery, elderly patients, prognostic biomarker

## Abstract

**Objective:** Postoperative cognitive dysfunction (POCD) is a common and severe complication of cardiovascular surgery. Lymphocyte-to-monocyte ratio (LMR) has been reported to be an independent predictor of lots of diseases associated with inflammation, but the association between the LMR and POCD is not clear. The present study aimed to investigate the potential value of LMR level to predict POCD in patients undergoing cardiovascular surgery.

**Methods:** A prospective observational study was performed on the patients diagnosed with heart diseases undergoing cardiovascular surgeries with cardiopulmonary bypass. The leukocyte counts were measured by blood routine examination preoperatively. Then we calculated the LMR by dividing the lymphocyte count by the monocyte count. Neurocognitive functions were assessed 1 day before and 7 days after surgery. Perioperative factors were recorded to explore the relationship between LMR and POCD.

**Results:** In total, 75 patients finished the whole study, while 34 patients developed POCD. The preoperative LMR level in the POCD group was higher than that in the non-POCD group. A cutoff value of 4.855 was identified to predict POCD occurrence according to ROC curve. The perioperative dynamic change of LMR level in the POCD group was higher than those in the non-POCD group. A cutoff value of 2.255 was identified to predict POCD occurrence according to ROC curve and the dynamic LMR change had similar varying trend with preoperative LMR level.

**Conclusions:** The dynamic change of LMR level in the peripheral blood is associated with occurrence of POCD, and preoperative LMR level seems to be a prognostic biomarker of postoperative cognitive dysfunction in patients after cardiovascular surgery.

## Introduction

Postoperative cognitive dysfunction (POCD) is a common and severe complication after cardiovascular surgery, especially in elderly patients (Ghaffary et al., 2015). POCD was defined as a significant reduction in cognitive performance after surgery, and diagnosed as decline in multiple neurocognitive domains, including memory, attention, coordination, orientation, verbal fluency, and executive function (Vacas et al., 2013), which would induce longer hospital stay, and increase mortality. The occurrence rate could be up to 40% 1 week after the cardiovascular surgery, and still as high as 17% 3 months after surgery (Rundshagen, 2014). Even worse, POCD could increase the long-term risk of Alzheimer's disease (AD) potentially (Bilotta et al., 2010; Hu et al., 2010). Therefore, more and more studies were performed to explore the underlying molecular mechanism and possible strategies for POCD prevention. However, the pathogenesis of POCD remains elusive.

In recent years, POCD has been reported to be associated with central immune inflammation, neuronal apoptosis, and synaptic dysfunction, etc. (Zhang et al., 2016; Liu and Yin, 2018; Wang et al., 2019). Notably, plenty of studies have shown that systemic inflammation was the major contributor to POCD, and that peripheral inflammatory cytokines caused by surgical trauma could enter the central nervous system via disruption of the blood-brain barrier (BBB) (Cibelli et al., 2010; Alam et al., 2018; Kotekar et al., 2018; Skelly et al., 2019). Multiple pro-inflammatory cytokines, such as IL-10 and IFN-α were extremely increased in the peripheral body fluid of patients and animal models (Wang et al., 2017, 2018; Zhu et al., 2018). To our knowledge, the activated leukocyte was an important component of innate immune response and its dynamic change could indicate the progress of disease. Moreover, it has been reported that counts of some leukocytes could reflect the status of systemic inflammation in various diseases, such as lymphocyte-to-monocyte ratio (LMR) and neutrophil-to-lymphocyte ratio (NLR) (Kose et al., 2019). And LMR has been shown to be a prognostic marker in a variety of cardiovascular diseases, coronary artery disease, ischemic stroke, as well as neurodegenerative disease (Gong et al., 2018; Kose et al., 2019; Lux et al., 2020; Umehara et al., 2020). However, the role of LMR to predict occurrence of POCD in patients undergoing cardiovascular surgeries has not been studied yet. Thus, identifying the LMR as a predictor of POCD is of importance.

Our study tried to explore the relationship between perioperative LMR level in the peripheral blood and occurrence of POCD, and investigate the ability of LMR level predicting POCD occurrence in patients undergoing cardiovascular surgeries.

## Materials and Methods

### Patients

The study protocol was approved by the West China Hospital of Sichuan University Biomedical Research Ethics Committee (Sichuan, China). The study was registered in the Chinese Clinical Trial Registry (ChiCTR-IPD-16008289). Patients or their relatives signed written informed consent before enrollment. The patients between September 2016 to December 2016 in West China Hospital undergoing cardiovascular surgeries with cardiopulmonary bypass were enrolled in this study. The inclusion criteria were (1) patients older than 50 years; (2) American Society of Anesthesiologists physical status I–III; (3) requiring an open heart surgery under general anesthesia; The exclusion criteria were (1) preoperative Mini-Mental State Examination (MMSE) score <24; (2) history of cardiothoracic surgery; (3) patients with central nervous system disease or psychiatric disease; (4) ejection fraction <40%; (5) patients who were refused or unable to complete the neurocognitive evaluation.

### Clinical Data Collection

On admission, demographic factors for instance, gender, age, body mass index (BMI), and education were collected, and a cardiologist examined the patients for the New York Heart Association classification. The possible perioperative confounding factors, such as the duration of CPB, duration of aorta cross-clamping, duration of surgery, duration of anesthesia, duration of ICU, and duration of hospitalization were recorded. Counts of hemocytes, hepatic, and renal function were measured by blood routine examination 1 day before and 7 days after surgery.

### Blood Collection

The blood samples were collected by a professional registered nurse 1 day before and 7 days after surgery, and the hematology analyzer (SYSMEXXN-10, Sysmex, Japan) was used to make the complete blood count in a clinical laboratory. We defined the lymphocyte-to-monocyte ratio as lymphocyte count divided by monocyte count. Preoperative dynamic change of the lymphocyte-to-monocyte ratio was defined as preoperative LMR minus postoperative LMR.

### Neurocognitive Evaluation

One day before and 7 days after surgery, a series of neurocognitive evaluation was performed by a trained investigator to access patients' cognitive function. Different cognition domains were assessed using several neuropsychological tests including: (1) Mini-Mental State Examination; (2) Word Immediate Recall Test, a test for short-term memory; (3) Image Immediate Recall Test, a test for immediate visuospatial and learning memory; (4) Trail Making Test A, a test for hand–eye coordination; (5) Digit Span Test, a test for concentration and attention; (6) Digit Symbol Coding Test, a test for psychomotor speed; (7) Word Delayed Recall Test, (8) Word Delayed Interference Test and (9) Image Delayed Recall Test, tests for long-term memory and delay recall abilities; (10) Image Delayed Interference Test and (11) Verbal Fluency Test, tests for fluency and executive function. Patients with severe cognitive impairment were excluded by MMSE evaluation preoperatively. We summarized the preoperative scores of all patients to obtain the standard deviation (SD) of each test. Then, all the postoperative worse performances were defined as negative change scores, and the absolute value of each change score was greater than the standard deviation (SD). A patient with two or more bad performances was considered to be in the POCD group. Otherwise, the patient was assigned to the non-POCD group. Besides, patients with two neuropsychological test defects were defined as mild POCD and patients with more than two neuropsychological test defects were defined as severe POCD.

### Anesthesia and Surgery

The same preoperative preparation was prepared for all the patients, including correction of electrolysis disorders, protection of cardiac function, injection of 3 mg scopolamine and 5 mg morphine preoperative, radial artery puncture for ambulatory blood pressure, central venous catheterization for central venous pressure, electrocardiogram monitoring, and oxygen saturation monitoring. We used midazolam (0.2–0.3 mg/kg), sufentanil (0.5–0.8 mg/kg), and cis-atracurium (2–3 mg/kg) for induction. We also used intravenous target control infusion to maintain propofol and remifentanil concentration, and injected midazolam, sufentanil, and cis-atracurium intermittently and intravenously for a stable anesthesia. We used an anesthesia machine to maintain mechanical ventilation, the respiratory rate was 12 times/min, and the tidal volume was 6–8 ml/kg. We performed central venous catheterization and detected the central venous pressure after anesthesia. During the cardiopulmonary bypass, we controlled the mean artery pressure at 50–80 mmHg, the perfusion dosage was 2.0–2.6 L/min/m^2^, the hematocrit was 25–30%, and the blood dilution was moderate. We used protamine to neutralize heparin in 1:1 ratio after the cardiopulmonary bypass. For further treatment, the patients were transferred into intensive care unit when the surgery finished.

### Statistical Analysis

All the continuous variables were presented as mean with standard deviation (SD) or median with interquartile range (IQR), and categorical variables were presented as a number with a percentage. Student *t*-test, Mann–Whitney U test, and Kruskal–Wallis test were used for comparisons between continuous variables, and chi-squared test or Fisher exact test were used for comparisons between categorical variables. Kolmogorov–Smirnov normality test and Bartlett test were used for the data homogeneity of variance test. Multivariate logistic regression was used to determine the independent predictors of POCD. The results were expressed as odds ratio (OR) and 95% confidence interval (95% CI). Potential risk factors, for instance, body mass index, age, duration of surgery, duration of CPB, duration of aorta cross-clamping, and duration of anesthesia were included in the multivariable logistic regression. The optimal threshold of LMR values for predicting POCD was determined by receiver operating characteristic (ROC) approach, while the area under the curve (AUC) and 95% CI were reported. All statistical analyses were performed with the IBM SPSS Statistical version 22.0 (IBM, Armonk, NY). A two-sided *P* < 0.05 was considered statistically significant. According to the sample size formula of one single population rate, n = ${\left(\frac{\mu }{\Delta }\right)}^{2}\times p\;\left(1-p\right)$ (*p* means population rate, *p* = 25%, Δ means allowable error, Δ = 0.1 and μ = 1.96), we calculate the minimum sample size is 72.

## Results

### Patients

**Figure 1:**
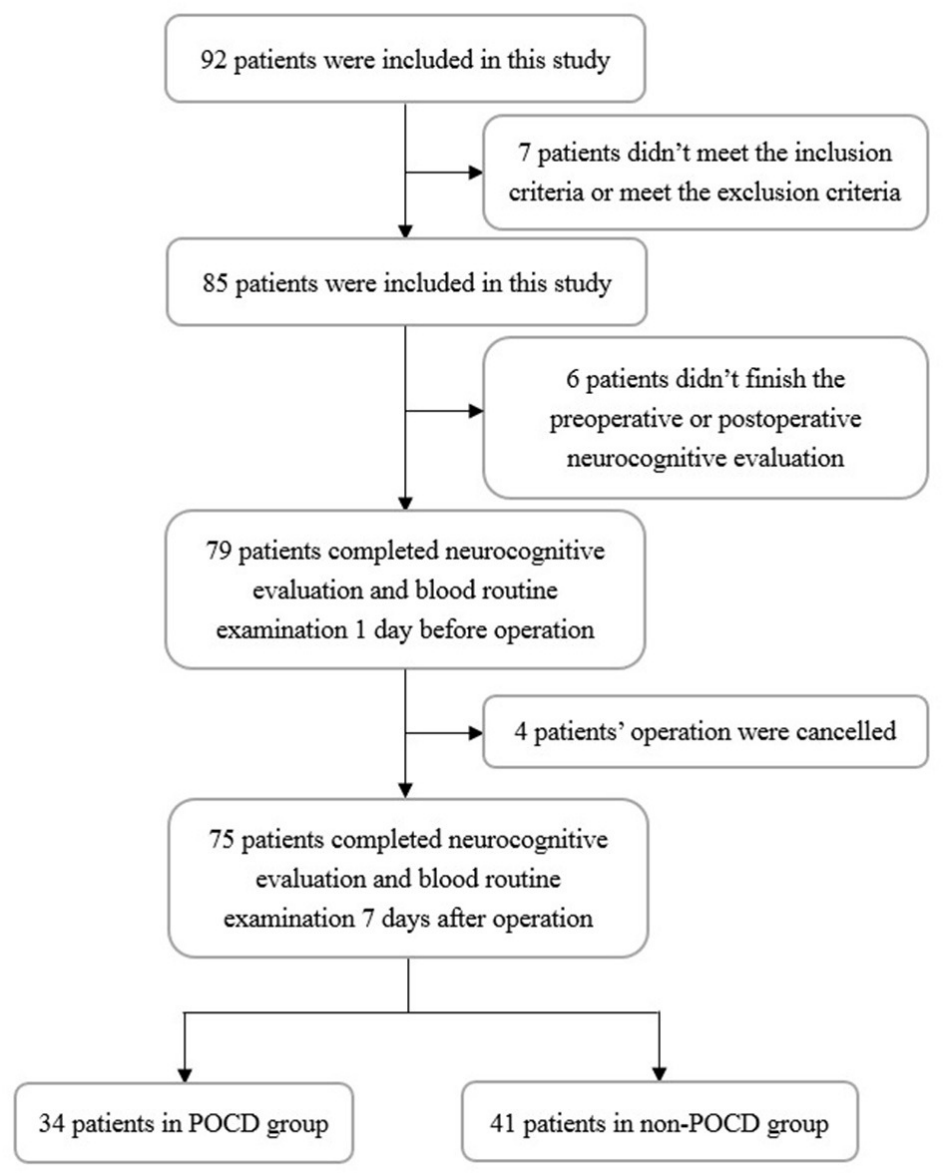
The process flow diagram of the experiment. POCD, postoperative cognitive dysfunction.

In total, 92 patients were included, and 75 patients finished the whole study (Figure 1). According to the neurocognitive evaluation, 34 (45.3%) patients were diagnosed as having postoperative cognitive dysfunction. All the baseline information and laboratory findings of the total cohort, both the POCD group and non-POCD group, are presented in Table 1. There was no difference between the two groups in gender, age, education level, body mass index, NYHA classification, cardiovascular risk factors (hypertension and diabetes), duration of anesthesia, operation, CPB and aortic cross-clamp, and hepatic and renal function. Additionally, there was no significant difference in the preoperative neurocognitive evaluations (Table 2) in the two groups. The POCD group showed poorer neuropsychological performance 7 days after surgery than non-POCD group, while there were significant differences in the Word Immediate Recall Test, Image Immediate Recall Test, Trail Making Test A, Digit Span Test, Word Delayed Recall Test, Word Delayed Interference Test, and Image Delayed Recall Test between the two groups (Table 3).

**Table 1:** Clinical features of the total cohort, POCD group, and non-POCD group.

	**Total cohort**	**POCD group**	**Non-POCD group**	
**Variables**	***n* = 75**	***n* = 34**	***n* = 41**	***P***
Age, y	58 (52.5–63)	59 (55–62.75)	57 (52–63.5)	0.743
Male, *n (%)*	37 (48.1)	16 (47.1)	21 (51.2)	0.649
BMI, kg/m^2^	23.37 (20.95–25.48)	23.56 (21.44–26.34)	23.21 (20.76–24.43)	0.236
Education (higher than middle school), *n (%)*	30 (39)	14 (41.1)	16 (39)	1.000
Hypertension, *n (%)*	16 (20.8)	8 (23.5)	8 (19.5)	0.786
Diabetes, *n (%)*	3 (3.9)	1 (2.9)	2 (4.9)	1.000
NYHA classification>II, *n (%)*	60 (77.9)	28 (82.3)	32 (78)	1.000
Duration of anesthesia, min	300 (270–320)	300 (262.5–320)	300 (280–330)	0.727
Duration of operation, min	235 (195–250)	231 (192.5–249.75)	240 (197.5–257.5)	0.503
Duration of CPB, min	125 (89–151.5)	114 (73.25–152.25)	133 (106–152)	0.071
Duration of aortic cross-clamp, min	84 (54.5–104.5)	79.5 (49–104)	84 (73–106)	0.366
Duration of ICU, d	3 (3–5)	4 (3–5)	3 (3–4)	0.105
Duration of hospitalization, d	9 (8–10.5)	9 (8–11)	9 (8–10)	0.333
ALT, U/L	21 (16–34.5)	19.5 (16–32.5)	21 (16–38)	0.585
AST, U/L	26 (21–32.5)	26.5 (22–32.75)	26 (20–32)	0.574
Platelet, × 10^9^/L	147 (105.5–185.5)	154.5 (115.5–189.5)	145 (103.5–171.5)	0.400
Serum creatinine, μmol/L	77 (67.65–87.3)	75.3 (66.75–84.25)	82 (67.65–90.1)	0.391
Preoperative monocyte	0.38 (0.31–0.44)	0.34 (0.28–0.43)	0.39 (0.34–0.46)	0.059
Postoperative monocyte	0.72 (0.61–0.98)	0.68 (0.56–0.95)	0.79 (0.66–0.99)	0.307
Preoperative lymphocyte	1.66 (1.28–2)	1.65 (1.39–1.87)	1.68 (1.23–2.22)	0.315
Postoperative lymphocyte	1.16 (0.89–1.56)	1.1 (0.89–1.66)	1.16 (0.88–1.55)	0.527
Preoperative LMR	4.43 (3.5–5.55)	5.28 (4.13–6.37)	4.13 (3.37–5.06)	0.001**
Postoperative LMR	1.55 (1.18–2.2)	1.8 (1.27–2.22)	1.37 (1.02–2.2)	0.111
ΔLMR	2.96 (1.82–4.07)	3.26 (2.32–4.23)	2.69 (1.64–3.58)	0.012*

Data are presented as median with IQR for continuous variables and as number with percentage for categorical variables. The P-value is calculated by the Mann-Whitney U test for continuous variables and by Fisher's exact test for categorical variables. P* means P-value < 0.05. P^**^ means P-value < 0.01. We diagnose the systolic pressure ≥40 mmHg and/or diastolic pressure ≥90 mmHg preoperative as hypertension. We diagnose the fasting blood glucose ≥7.0 mmol/L and/or postprandial blood glucose ≥11 mmol/L preoperative as diabetes. POCD, postoperative cognitive dysfunction; BMI, body mass index; NYHA, New York Heart Association; CPB, cardiopulmonary bypass; ICU, intensive care unit; ALT, Alanine aminotransferase; AST, Aspartate aminotransferase; LMR, Lymphocyte-to-Monocyte Ratio; ΔLMR, preoperative LMR minus postoperative LMR.

**Table 2:** Preoperative cognitive functions of total cohort, POCD group, and non-POCD group.

	**Total cohort**	**POCD group**	**Non-POCD group**	
**Tests**	***n* = 75**	***n* = 34**	***n* = 41**	***P***
Mini-mental state examination	27 (25–29)	27 (25–29)	27 (25–28.5)	0.660
Word immediate recall test	12 (9–15)	13 (10–16)	11 (9–15)	0.194
Image immediate recall test	8 (5–11)	8 (6.25–10.75)	8 (5–11.5)	0.798
Trail making test A	62 (47.28–82.57)	66.77 (49.11–83.27)	62.61 (47.05–82)	0.469
Digit span test	15 (12–17)	14.5 (12–17)	16 (12.5–18)	0.411
Digit symbol coding test	28 (18.5–33)	29 (18.5–35)	28 (18.5–32)	0.834
Word delayed recall test	3 (1–4)	3 (1.25–5)	2 (1–3)	0.171
Word delayed interference test	20 (18–22)	21 (18–23)	20 (17–21)	0.084
Image delayed recall test	3 (2–5)	3.5 (2–5)	3 (2–5)	0.648
Image delayed interference test	11 (10–12)	10.5 (9.25–12)	11 (10–12)	0.267
Verbal fluency test	36 (28–44)	39.5 (27.5–47)	33 (28–40)	0.119

*Data are presented as median with IQR. The P-value is calculated by the Mann-Whitney U test. POCD, postoperative cognitive dysfunction*.

**Table 3:** Postoperative cognitive functions of total cohort, POCD group, and non-POCD group.

	**Total cohort**	**POCD group**	**Non-POCD group**	
**Tests**	***n* = 75**	***n* = 34**	***n* = 41**	***P***
Mini-mental state examination	27 (24–28)	27 (23–28)	27 (25–29)	0.343
Word immediate recall test	12 (8–14)	9 (7–13)	12 (10–14)	0.029*
Image immediate recall test	6 (3–11)	5 (2–6)	10 (5–13)	0.001**
Trail making test A	59.87 (44.36–77.15)	61.83 (50.89–96.97)	57.41 (40.74–75.1)	0.068
Digit span test	15 (12–18)	14 (12–16)	16 (13–19)	0.017*
Digit symbol coding test	26 (18–32.5)	24.5 (15.25–29.75)	27 (19.5–35)	0.041*
Word delayed recall test	2 (1–4)	1.5 (0–3)	3 (1–5)	0.010*
Word delayed interference test	19 (15–20)	18 (15–20)	19 (18–20.5)	0.063
Image delayed recall test	2 (1–4.5)	2 (1–3)	4 (2–5)	0.001**
Image delayed interference test	11 (9–12)	10 (8–11)	11 (9.5–12)	0.041*
Verbal fluency test	29 (23–35)	30 (23–35)	28 (22–34)	0.510

Data are presented as median with IQR. The P-value is calculated by the Mann-Whitney U test. P* means P-value < 0.05. P^**^ means P-value < 0.01. POCD, postoperative cognitive dysfunction.

### Association Between LMR and POCD Occurrence

**Figure 2:**
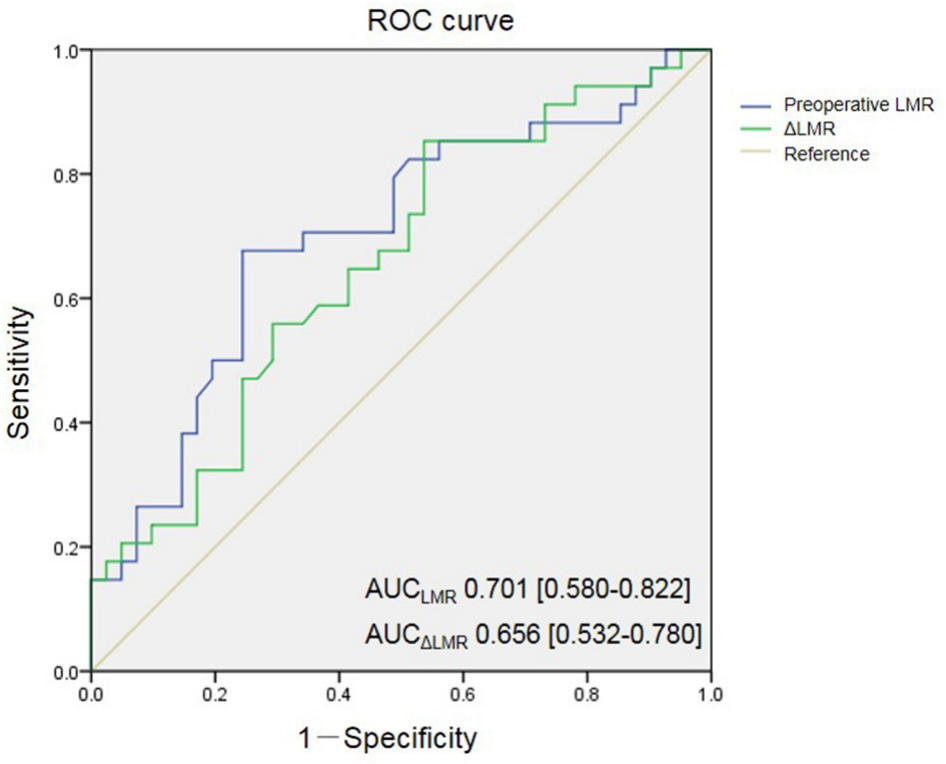
Receiver-operating characteristic (ROC) curves for preoperative LMR and change of LMR. AUC indicates area under curve.

**Figure 3:**
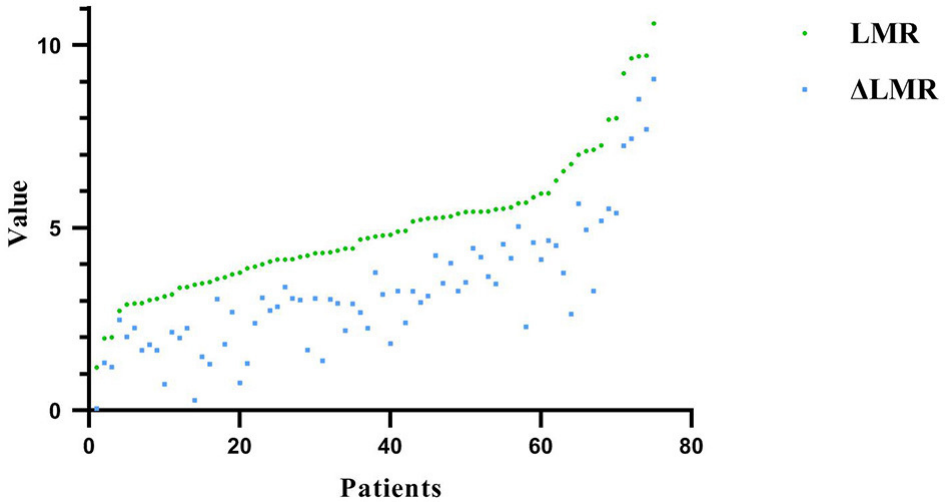
Preoperative LMR has a similar varying trend with change of LMR.

The preoperative LMR level in the POCD group was significantly higher than that of the non-POCD group (*P* = 0.001, Table 1). Besides, we found that the preoperative dynamic change of the LMR level, the preoperative LMR minus postoperative LMR, in the POCD group was significantly higher than that of the non-POCD group (*P* = 0.012, Table 1). In order to find the most appropriate biomarker value to predict the occurrence of POCD, preoperative LMR level and perioperative LMR dynamic change were investigated. Results of the multivariable logistic regression analysis showed that LMR and perioperative LMR dynamic change were independently associated with POCD (Supplementary Table 1). According to ROC curve, a cutoff value of 4.855 (specificity: 0.756, sensitivity: 0.676) of preoperative LMR level could be used to predict POCD occurrence and AUC was 0.701 (95% CI, 0.580–0.822, *P* = 0.003). Meanwhile, a cutoff value of perioperative LMR change of 2.255 (specificity: 0.463, sensitivity: 0.853) was also analyzed to predict POCD occurrence and AUC was 0.656 (95% CI, 0.532–0.780, *P* = 0.02) (Figure 2). Besides, we found that preoperative LMR had similar varying trend with LMR dynamic change (Figure 3). Then, we divided the patients into two groups based on the cutoff value of preoperative LMR, the High-LMR group and the Low-LMR group (LMR > 4.855 and LMR ≤ 4.855, respectively). There was a significant higher occurrence of POCD in the High-LMR group compared with the Low-LMR group (*P* < 0.001, Table 4). Moreover, there was no difference between the mild and severe POCD group for the preoperative LMR value and perioperative change value of LMR (Supplementary Table 2).

**Table 4:** Clinical features of High-LMR group and Low-LMR group.

	**High-LMR group (LMR > 4.855)**	**Low-LMR group (LMR ≤ 4.855)**	
**Variables**	***n* = 33**	***n* = 42**	***P***
LMR at diagnosis	5.69 (5.41–7.12)	3.68 (3.03–4.29)	0.000***
Age, y	58 (55–61)	58.5 (51.25–64)	0.726
Male, *n (%)*	13 (39.4)	24 (57.1)	0.250
BMI, kg/m^2^	23.21 (20.3–25.49)	23.39 (21.16–25.5)	0.711
Diagnose as POCD, *n (%)*	23 (69.7)	11 (26.2)	0.001**
Education (higher than middle school), *n (%)*	16 (48.5)	14 (33.3)	0.162
Hypertension, *n (%)*	6 (18.2)	10 (23.8)	0.778
Diabetes, *n (%)*	2 (6.1)	1 (2.4)	0.573
NYHA classification>II, *n (%)*	26 (78.8)	34 (80.9)	1.000
Duration of anesthesia, min	300 (272.5–327.5)	300 (262.5–310)	0.370
Duration of operation, min	235 (195–254)	235.5 (193.75–250)	0.947
Duration of CPB, min	122 (87–148.5)	128.5 (102.7–153.7)	0.647
Duration of aortic cross-clamp, min	85 (51.5–105.5)	84 (58.25–104.5)	0.938
Duration of ICU, d	4 (3–5)	3 (3–4)	0.547
Duration of hospitalization, d	9 (8–11)	9 (8–10)	0.817
ALT, U/L	19 (15.5–34.5)	21 (16–34.75)	0.621
AST, U/L	26 (22.5–31)	26.5 (19.5–33.75)	0.885
Platelet, × 10^9^/L	145 (101–189)	148.5 (107.2–177.5)	0.902
Serum creatinine, μmol/L	75 (65.5–90.65)	82 (69.25–87.45)	0.234
Monocyte, × 10^9^/L	0.33 (0.265–0.41)	0.39 (0.34–0.5)	0.002**
Lymphocyte, × 10^9^/L	1.9 (1.65–2.35)	1.44 (1.17–1.8)	0.000***
Neutrophil, × 10^9^/L	3.2 (2.63–3.87)	3.67 (2.67–4.33)	0.141

Data are presented as median with IQR for continuous variables and as number with percentage for categorical variables. The P-value is calculated by the Mann-Whitney U test for continuous variables and by Fisher's exact test for categorical variables. P^**^ means P-value < 0.01. P^***^ means P-value < 0.001. We diagnose the systolic pressure ≥140 mmHg and/or diastolic pressure ≥90 mmHg as hypertension. We diagnose the fasting blood glucose ≥7.0 mmol/L and/or postprandial blood glucose ≥11 mmol/L as diabetes. POCD, postoperative cognitive dysfunction; BMI, body mass index; NYHA, New York Heart Association; CPB, cardiopulmonary bypass; ICU, intensive care unit; ALT, alanine aminotransferase; AST, aspartate aminotransferase; LMR, Lymphocyte-to-Monocyte Ratio.

Data are presented as median with IQR for continuous variables and as number with percentage for categorical variables. The P-value is calculated by the Mann-Whitney U test for continuous variables and by Fisher's exact test for categorical variables. P^*^ means P-value < 0.05. P^**^ means P-value < 0.01. P^***^ means *P*-value < 0.001. We diagnose the systolic pressure ≥140 mmHg and/or diastolic pressure ≥90 mmHg as hypertension. We diagnose the fasting blood glucose ≥7.0 mmol/L and/or postprandial blood glucose ≥11 mmol/L as diabetes. POCD, postoperative cognitive dysfunction; BMI, body mass index; NYHA, New York Heart Association; CPB, cardiopulmonary bypass; ICU, intensive care unit; ALT, alanine aminotransferase; AST, aspartate aminotransferase; LMR, Lymphocyte-to-Monocyte Ratio.

## Discussion

POCD is a severe nervous systematic complication of cardiovascular surgery and rating scales are commonly used for the diagnosis of POCD. However, the rating scale was not widely carried out for its time-consuming and laborious process (Rasmussen et al., 2001). Moreover, most aged patients undergoing cardiovascular surgery are in poor conditions and the scale results are always disturbed by their un-cooperation for pain, depression and hearing disorders (Hogan et al., 2013). Thus, it is of importance to find a biomarker to predict the occurrence of POCD easily. In our study, we found that there was an association between the LMR and occurrence of POCD, and the association remains when accounting for the common risks, such as age, perioperative factors, education etc. The preoperative LMR and perioperative LMR change had similar varying trends and they were both independent risks for predicting POCD occurrence.

Previous studies have demonstrated that leucocytes are cellular components of systemic inflammation (Cunningham, 2013). Both lymphocytes and monocytes play important roles in the immune regulatory pathway (Moens et al., 2008). The LMR is a combination of two independent markers of inflammation and was reported to be a potential novel biomarker of inflammatory response (Umehara et al., 2020). Gary et al. (2014) found that in patients with peripheral arterial occlusive disease, the decreasing of LMR is associated with a high risk of critical limb ischemia and other vascular endpoints. Besides, decreases in LMR have been used as biomarkers to predict outcomes in many diseases (Rajwa et al., 2018; Li et al., 2019; Russell et al., 2019). However, there are few studies on this topic in association with POCD, although the relationship between inflammation and the pathogenesis of POCD has long been discussed. In the current study, we found the surgery process indeed increased monocyte and decreased lymphocyte quantities in all patients. Nevertheless, the dynamic perioperative change of LMR in the POCD group was significantly higher than that in the non-POCD group. It has been reported that the decrease of the LMR level indicates that the patients are undergoing a state of systemic inflammation and POCD occurs when the inflammation level changes to a certain extent (Liu and Yin, 2018). Thus, the dynamic change of inflammatory biomarkers may reflect the balance between inflammation and immune response more accurately. Previous studies of neurodegeneration diseases have demonstrated that a decrease in LMR is linked to systemic inflammation, which could transform to neuroinflammation (Umehara et al., 2020). In a study with 162 patients diagnosed with advanced non-small-cell lung cancer, Sekine et al. (2018) reported that a change of the LMR level in patients was associated with the nivolumab monotherapy effect. As we know, the immune system plays an important role in controlling and eradicating tumor cells, and the dynamic change of LMR level reflects the efficacy of nivolumab therapy (Sekine et al., 2018). Thus, the perioperative change of the LMR level seems to be a prognostic biomarker of inflammation response. Moreover, we found that the preoperative LMR have similar varying trends with the change of LMR and it enabled the preoperative LMR level to be a preoperative predictive factor for the occurrence of POCD in patients undergoing cardiovascular surgery.

Systemic inflammation is closely associated with the occurrence and progression of POCD. A previous study has shown that an overly strong immune response with a cytokine storm may cause a complex state resulting in multiple organ dysfunction (Wang and Ma, 2008). Lots of animal studies supported the hypothesis that peripheral surgical trauma and insistent inflammatory status could cause central nervous functional disruption via the blood-brain barrier breakdown leading to POCD (Wang et al., 2017, 2018; Zhu et al., 2018). Rising monocytes play an important role in this progress in that they could bind to the damage-associated molecular patterns released from surgical sites through their membrane receptors in response to the damaged cells (Lotze and Tracey, 2005; Zhang et al., 2010). Moreover, the monocytes could present in the central neural system while the BBB was breaking down and they continued to secrete pro-inflammatory cytokines via upregulation of nuclear factor kappa B (NF-κB) expression, which may lead to a long period of cognitive decline (Saxena and Maze, 2018). Besides, a large amount of lymphocyte apoptosis was found in response to the inflammatory stress and decreased circulating lymphocytes might indicate a poor outcome in systemic inflammatory disease (Girardot et al., 2017). Liu and colleagues reported that lymphocyte counts were decreased in AD, and the underlying mechanism might be that the microglia could release TNF-α, which recruited lymphocytes from peripheral circulation across the impaired BBB into the central nervous system (Liu et al., 2010). Consistently, our results showed that postoperative monocytes were increased and lymphocytes were decreased in all patients and an obvious perioperative dynamic change of LMR was found in POCD patients. Thus, we supposed increased LMR perioperative change and a higher preoperative LMR value could predict the occurrence of POCD on the association of LMR related inflammation.

Until now, early recognition, early prevention, and management of perioperative risk factors seem to be the best modality to deal with POCD. Instead of postoperative biomarkers, a valuable preoperative biomarker from the patient, such as the high-LMR, could warn about the potential risk of the occurrence of POCD in advance. Additionally, a higher preoperative LMR value could let the surgeons, anesthesiologists, and nurses deal with the perioperative risk factors preparedly, which might even affect the operation and anesthesia plan. For example, more careful pre-anesthesia medication selection, closer monitoring of intraoperative anesthesia depth, and precise control of temperature and fluid management might let the patients avoid the occurrence of disease and return to family and society as soon as possible (Kotekar et al., 2018). Our results showed that the occurrence of POCD in patients who underwent cardiovascular surgery with cardiopulmonary-bypass is 45.3%, which was similar to the previous study (Norkiene et al., 2010). In our study, only the elderly patients were included and Luo and colleagues reported that the integrity of the BBB would be lost during the normal aging process. Aging related microglia was also associated with the neuroinflammation and synaptic aberration which may contribute to POCD (Luo et al., 2019). Meanwhile, Hovens and colleagues reported an animal research that cardiovascular surgery could affect more encephalic regions than non-cardiovascular surgery, including the hippocampus, hypothalamus, and prefrontal cortex. And ischemia-reperfusion injury was inevitable in cardiovascular surgery, which may lead to systemic inflammatory response and postoperative myocardial damage (Hovens et al., 2016). Moreover, there was no significant difference in demographic characteristics, cardiovascular risk factors, and perioperative factors between the POCD and non-POCD group in our current study. Consistently, Soenarto et al. (2018) have reported that the duration of cross-clamp, the duration of cardiopulmonary bypass, diabetes mellitus, and education level were not significantly different in the POCD and non-POCD group. However, Vacas et al. (2013) reported that the duration of surgery and anesthesia and BMI would be the potential risk factors for POCD, which might be due to the different populations and neurocognitive testing modalities. There are also some limitations in our study. First, the sample size of this study was small, with 75 patients finishing the whole study. Second, for a more complete assessment, we used a series of neurocognitive evaluation to test patients' cognitive function, which may be different from some other studies. Third, we only included the patients with cardiopulmonary bypass surgery and other types of surgery should be further investigated.

## Conclusion

We found that dynamic decreases in the LMR level was associated with poor neurocognitive function among the patients undergoing cardiovascular surgery, and preoperative LMR had similar varying trend with LMR dynamic change. Therefore, patients with higher preoperative LMR level should be more careful with taking anesthesia, operation, and postoperative care. Due to the low price and easy examination, LMR level seems to be a valuable prognostic biomarker. Therefore, the LMR level in the peripheral blood seems to be a biomarker for the prognosis of POCD patients after cardiovascular surgery. More investigations, especially large sample clinical studies, are needed to verify our findings and clarify the potential mechanisms of LMR in patients with POCD.

## Data Availability Statement

The raw data supporting the conclusions of this article will be made available by the authors, without undue reservation.

## Ethics Statement

The studies involving human participants were reviewed and approved by ChiCTR-IPD-16008289. The patients/participants provided their written informed consent to participate in this study.

## Author Contributions

QZ, RG, and CL designed the experiments. QZ, RG, CL, HC, and XZ performed the experiments. QZ, RG, CL, YQ, XC, and PL analyzed the data. QZ, RG, CL, JG, XX, CC, and TZ wrote the main manuscript text. All the authors reviewed the manuscript.

## Conflict of Interest

The authors declare that the research was conducted in the absence of any commercial or financial relationships that could be construed as a potential conflict of interest.
