# Supplementary material for: Dynamic Change of Lymphocyte-to-Monocyte Is Associated With the Occurrence of POCD After Cardiovascular Surgery: A Prospective Observational Study
Source: Front Behav Neurosci. 2021 Apr 13;15:646528. doi: 10.3389/fnbeh.2021.646528 (PMC8076514; doi:10.3389/fnbeh.2021.646528)
Supplement: Supplementary file 1 [file Data_Sheet_1.docx]

Supplementary Table 1. Result of multivariate logistic regression analysis

|  | OR | OR 95% CI | *P* value |
| --- | --- | --- | --- |
| LMR | 1.635 | (1.186-2.254) | 0.003^**^ |
| ΔLMR | 1.501 | (1.102-2.045) | 0.01^*^ |
| Duration of anesthesia | 1.000 | (0.991-1.010) | 0.922 |
| Duration of operation | 0.998 | (0.989-1.008) | 0.666 |
| Duration of CPB | 0.992 | (0.981-1.004) | 0.200 |
| Duration of aortic cross-clamp | 0.994 | (0.980-1.009) | 0.454 |
| Age | 1.010 | (0.938-1.087) | 0.792 |
| BMI | 1.108 | (0.953-1.288) | 0.183 |

*P*^*^ means *P* value <0.05. *P*^**^ means *P* value <0.01. OR, odds ratio; CI, confidence interval; LMR, Lymphocyte-to-Monocyte Ratio; ΔLMR, preoperative LMR minus postoperative LMR.

Supplementary Table 2. Clinical features of mild group and severe group

|  | Mild group | Severe group |  |
| --- | --- | --- | --- |
| Variables | n=16 | n=18 | *P* |
| LMR at diagnosis | 5.44 (4.57-6.12) | 5.29 (4.13-7) | 0.827 |
| ΔLMR | 3.46 (2.675-4.64) | 3.26 (2.4-4.59) | 0.661 |
| Age, y | 59 (53-63) | 59 (55-62) | 1.000 |
| Male, n (%) | 9 (56.3) | 7 (38.9) | 0.503 |
| BMI, kg/m^2^ | 23.63 (21.57-24.97) | 23.37 (20.25-27.03) | 0.876 |
| Education (higher than middle school), n(%) | 7 (43.7) | 7 (38.9) | 1.000 |
| Hypertension, n(%) | 7 (43.7) | 1 (5.5) | 0.016^*^ |
| Diabetes, n(%) | 0 (0) | 1 (5.5) | 1.000 |
| NYHA classification>Ⅱ, n(%) | 13 (81.3) | 15 (83.3) | 1.000 |
| Duration of anesthesia, min | 300 (270-330) | 300 (260-300) | 0.346 |
| Duration of operation, min | 237 (202.5-254) | 230 (190-240) | 0.175 |
| Duration of CPB, min | 114 (80-153) | 104 (70-135) | 0.433 |
| Duration of aortic cross-clamp, min | 90 (51-116) | 67 (49-104) | 0.471 |
| Duration of ICU, d | 4 (3-5) | 4 (3-5) | 0.661 |
| Duration of hospitalization, d | 9 (8-10.5) | 9 (8-12) | 0.471 |
| ALT, U/L | 19 (15.5-34.5) | 21 (16-34.75) | 0.621 |
| AST, U/L | 26 (21.5-34.5) | 27 (22-31) | 0.802 |
| Platelet, ×10^9^/L | 124 (111-184.5) | 171 (117-205) | 0.244 |
| Serum creatinine, μmol/L | 75.6 (64.5-90) | 75 (69-82) | 1.000 |
| Monocyte, ×10^9^/L | 0.34 (0.28-0.42) | 0.35 (0.24-0.43) | 0.950 |
| Lymphocyte, ×10^9^/L | 1.67 (1.44-2.02) | 1.68 (1.42-2.31) | 0.950 |
| Neutrophil, ×10^9^/L | 3.36 (2.64-3.89) | 3.36 (2.64-3.94) | 0.684 |

Data are presented as median with IQR for continuous variables and as number with percentage for categorical variables. The P value is calculated by the Mann-Whitney U test for continuous variables and by Fisher’s exact test for categorical variables. *P*^*^ means *P* value <0.05. We diagnose the systolic pressure ≥140mmHg and/or diastolic pressure ≥90mmHg as hypertension. We diagnose the fasting blood glucose ≥7.0mmol/L and/or postprandial blood glucose ≥11mmol/L as diabetes. POCD, postoperative cognitive dysfunction; BMI, body mass index; NYHA, New York Heart Association; CPB, cardiopulmonary bypass; ICU, intensive care unit; ALT, alanine aminotransferase; AST, aspartate aminotransferase; LMR, Lymphocyte-to-Monocyte Ratio.
